# Preference for Cute Infants Does Not Depend on Their Ethnicity or Species: Evidence from Hypothetical Adoption and Donation Paradigms

**DOI:** 10.1371/journal.pone.0121554

**Published:** 2015-04-06

**Authors:** Jessika Golle, Fabian Probst, Fred W. Mast, Janek S. Lobmaier

**Affiliations:** 1 Department of Psychology, University of Bern, Bern, Switzerland; 2 Center for Cognition, Learning, and Memory, University of Bern, Bern, Switzerland; 3 Hector Research Institute of Education Sciences and Psychology, University of Tübingen, Tübingen, Germany

## Abstract

Results of previous work suggest a preference of adult observers for cute compared to less cute infants. In Study 1 we investigated whether the preference for cute infants depends on the ethnicity and species of the infant. We simultaneously presented two faces (one cute and one less cute) and asked Caucasian participants to choose the infant to whom they would rather give a toy (Task 1) and which infant they would rather adopt (Task 2). The infants were Caucasian or African human babies or dog puppies. For all face categories and in both tasks we found a strong preference for cute infants. A possible reason for preferring cute infants may be that cute infants look healthier than less cute infants. To investigate whether cuteness is associated with the assessment of health we conducted Study 2. Faces of Caucasian and African infants and dog puppies were rated for cuteness and health. The findings revealed a significant relationship between health and cuteness evaluation across all stimuli. We suggest that one reason why cute infants are preferred might be because they are perceived as being healthier.

## Introduction

Infants of many species are born helpless and depend on protection and care. According to Lorenz [1], the Kindchenschema is an innate releasing mechanism for caretaking behavior and affective orientation toward infants. This schema is triggered by paedomorphic features such as a relatively large head compared to the size of the body, a relatively big cranium compared to the facial bones, large eyes that lie below the horizontal midline of the skull, a soft-elastic surface texture, and round protruding cheeks. Infants that conform to this “babyfacedness” are commonly described as being cute [e.g. 2, 3]. These infants are not only perceived as looking cute, they are also rated as being likable, sociable, smart, competent, easy to care for, and good [4, 5]. The association of cuteness with various positive attributes can be described by the cute-is-good stereotype [5] derived from the beautiful-is-good stereotype [5, 6]. The cute-is-good stereotype describes a general preference for cute infants: They are liked the best [7], motivation to care for them is higher [2], they experience more affectionate interactions with their mothers [8, 9], get fewer punishments [10, 11], and they probably get different treatments from nurses that positively affect infants’ physical wellbeing [cf. 12].

Lorenz originally described the features triggering the Kindchenschema in humans independent of species. As yet, empirical evidence of cross-species and cross-ethnicity effects of cuteness on observer preferences and behaviors is relatively scarce. Most of the existing studies investigated cuteness perception within one ethnicity [e.g. 2, 10, 13–17] or did not consider ethnicity as a separate variable in their analyses [e.g. 3, 4, 18, 19, 20].

Only a few studies focused on cross-ethnicity [21, 22] and cross-species effects of facial cuteness on observer preferences and behavior [23–27]. Empirical findings suggest that the same facial features are associated with cuteness across ethnicities. Chin et al. [21] found that infants with large eyes are perceived to be cuter than infants with small eyes, irrespective of the infants’ ethnicity. The attribution of positive traits on cute infants is also consistent across ethnicities [5, 28]. Golle et al. [23] even found evidence that cuteness perception of different species involves at least some common coding mechanism. A further study found that babyish shape-related features of a human infant alter the evaluation of cuteness of a cat [24]. Cuteness not only influences preference or the attribution of positive traits, it also alters behavior [25, 26]. Both Nittono et al. [25] and Sherman et al. [26] found more careful behavior of human participants in an operation game after viewing pictures of baby animals compared to after viewing pictures of adult animals.

A possible explanation for these positve effects of cute infants across ethnicity and species on trait attributions and behavior is that cuteness implies reduced breeding costs because cuteness might be associated with health and good genes [16, 22, 29–33]. Previous studies found that cues of Down’s syndrome, fetal alcohol syndrome [29, 30], low body weight, and high body weight [33] alter facial cues and influence ratings of health and cuteness. Healthy infants need less care than infants that are sickly or seriously ill. To the best of our knowledge only few studies have investigated the relationship between perceived cuteness and health within and across faces of different ethnicities [16, 22] and species [27]. These studies found a positive association between health and cuteness.

None of the above-mentioned studies compared cuteness preferences for faces of different ethnicities and species at the same time. Nor has any study analyzed the role of health perception in cuteness evaluations across infants of different ethnicities and species. In the current study we investigated whether male and female Caucasian observers of different ages generally prefer cute faces of African and Caucasian infants and dog puppies, and whether the perception of cuteness is related to the perception of health status across infants of different ethnicities and species. In Study 1 we used a two alternative forced choice (2AFC) paradigm in which two faces were presented simultaneously (one cute and one less cute). We asked participants to choose the infant to whom they would rather give a toy (hypothetical donation task) and to choose the infant which they would rather adopt (hypothetical adoption paradigm: HAP [16, 30]). The stimuli consisted of either Caucasian or African infants, or dog puppies. By presenting faces of different ethnicities *and* species we extend on previous studies [16, 22, 30] to further investigate whether Caucasian observers generally prefer cute over less cute infants of their own as well as of different ethnicity and species.

In Study 2, we investigated whether perceived health and cuteness are associated for faces of different ethnicities and species. In one block we asked participants to rate how often the infant might be ill. In a second block we asked participants to rate the faces for cuteness. Following previous studies we used faces of the same ethnicity as the observer [16] as well as faces of a different ethnicity [22] and species [27], and we directly compared the association between cuteness and health for faces of all stimulus classes.

If cuteness is a universal concept that automatically triggers care-taking behavior, we expect that cute infants should be preferred over less cute infants regardless of ethnicity or species. Specifically, cute infants of all ethnicities and species should be more likely to receive a toy and more likely to be adopted. We further predict a negative association between perceived cuteness and how often the infant might be ill, for Caucasian and African infants as well as for puppy faces.

## Pre-Study: Cuteness Rating

In order to establish a set of cute and less cute infant faces for Study 1 we asked 24 Caucasian participants to rate the cuteness of African and Caucasian infant and dog puppy faces. Cuteness ratings are a good indicator of perceived cuteness and observers highly agree on which faces they find cute [cf. 15, 23, 34, 35].

### Methods

#### Participants

Twelve male and 12 female students of the University of Bern ranging in age between 20 and 29 years (*M* = 24, *SD* = 2.8) participated in this rating study. All perticipants reported to be of Caucasian descent. They gave verbal informed consent and they were told that they could abort the experiment at anytime without giving any reasons. The research and consent procedure was approved by the ethics committee of the Faculty of Human Sciences of the University of Bern and conformed with the “Ethical Principles of Psychologists and Code of Conduct” of the American Psychological Association [36].

#### Stimuli

Pictures of 100 Caucasian and 100 African infant and 100 dog puppy faces were collected from different sources (internet, personal collections) and showed natural, colored, non-manipulated infant faces with a neutral facial expression. Because many pictures were collected from the internet we do not have accurate age and sex information for all images. All stimuli had a size of 400 x 400 pixels and appeared at a resolution of 124.75 PPI. Participants were seated 60 cm in front of a 12.1” computer screen with a resolution of 1280 x 800 pixel, subtending a visual angle of 7.8° x 7.9°.

#### Procedure

Pictures were presented in the center of the computer screen until the participant responded. The task was to assess the cuteness of each infant face on a 7-point Likert-like rating scale (1 = not cute at all to 7 = very cute). Caucasian and African infant and dog puppy faces were presented block-wise per stimulus category. Block order was randomly assigned.

### Results

The inter-rater reliability of these ratings was very high (Cronbach’s Alpha = 0.94, ICC = 0.93). Based on the rating scores of the pre-study, 40 stimuli (20 cute, 20 less cute) of each face category (African, Caucasian, dog puppies) were selected for Study 1. We paired a cute with a less cute face so that the two faces in each pair differed clearly in cuteness. This was done to enhance the probability that the difference between cute and less cute infant faces could be easily perceived. The cuteness differences in the face pairs did not differ across categories. The mean cuteness scores of all stimuli as well as the mean cuteness score differences across all face pairs and their ranges (Min and Max) are shown in Table 1.

**Table 1:** Mean cuteness scores of the stimuli used in Study 1.

	cute	less cute	Mean cuteness difference	Min	Max
Caucasian infants	5.5	2.9	2.6	2.5	2.7
African infants	5.5	2.9	2.5	2.1	3.1
dog puppies	5.5	3.0	2.5	2.2	3.3

Using multilevel regression analyses for repeated measurements [37], we tested the assumption that the selected 20 cute faces were rated to look significantly cuter than 20 less cute faces for each stimulus category and that this difference should not vary between face categories. Data was analyzed with respect to within-subject and between-subject variation without aggregating cuteness rating scores (commonly used in variance analysis procedures). The dependent variable was the cuteness rating score. To judge whether a multilevel model is warranted the random intercept model without any predictor was calculated and revealed significant between-subject variance, s^2^(*β*_0t_) 0.26, *z* = 3.07, *SE* = 0.09, *p* = .002. The intra class correlation was 0.08, calculated with the following formula: s^2^(*β*_0t_) / s^2^(*β*_0t_) + s^2^(ε_*it*_), s^2^(ε_*it*_) = 3.25, *z* = 37.79, *SE* = 0.09, *p* < .001 [38, 39]. Level 1 units are the stimuli and Level 2 units are the participants. Thus, predictors on Level 1 are equivalent to within-subject factors (cf. variance analysis strategies) and Level 2 predictors are equivalent to between-subject factors. In the pre- study only within-subject factors were relevant. In Model 1 the predictors were two Indicator variables (Dummy variables) reflecting whether the faces were Caucasian infants or not (*I*_*1it*_: African = 1, Caucasian = 0, dog puppies = 0; *I*_*2it*_: African = 0, Caucasian = 0, dog puppies = 1), cuteness category (*C*_*it*_: 0 = less cute, 1 = cute), and interactions between cuteness category and the two Indicator variables (see Equation 1).

$$\begin{array}{c} Y_{it}=\beta_{0t}+\beta_{1t}I_{1it}+\beta_{2t}I_{2it}+\beta_{3t}C_{it}+\beta_{4t}I_{1it}C_{it}+\beta_{5t}I_{2it}C_{it}+\varepsilon_{it}\\ \forall \beta_{jt}\left(j=0,\ldots ,3\right):\beta_{jt}=\alpha_{j0}+\upsilon_{jt} \end{array}$$ (1)

Because there were no significant interaction terms and no significant Indicator variables the fixed intercept indicates that the expected cuteness value for less cute infant faces was 2.92, *z* = 20.51, *p* < .001. The significant cuteness category coefficient indicates that cute faces were assessed cuter than less cute faces (mean difference between cute and less cute faces = 2.59, *z* = 14.72, *p* < .001). We found significant differences between cute and less cute infant and puppy faces and there was no differential effect for face category (African, Caucasian, dog puppies). See Table 2 for more details.

**Table 2:** Results of the pre-study (Model 1).

	Coefficient	*z*	*SE*	*p*
Fixed effects				
*Level 1*				
Intercept	2.92	20.51	0.14	<.001
*I*_1*it*_	-0.01	-0.07	0.11	.941
*I*_2*it*_	0.10	0.68	0.15	.496
*C*_*it*_	2.59	14.72	0.18	<.001
*I*_1*it*_ *C*_*it*_	-0.05	-0.38	0.13	.701
*I*_2*it*_ *C*_*it*_	-0.17	-0.94	0.18	.350
Random effects				
*s* ^2^(*ε*_*it*_)	1.49	37.30	0.04	<.001
*s* ^2^(*υ*_0*t*_)	0.41	3.02	0.13	.003
*s* ^2^(*υ*_1*t*_)	0.16	2.32	0.07	.020
*s* ^2^(*υ*_2*t*_)	0.20	2.45	0.08	.014
*s* ^2^(*υ*_3*t*_)	0.45	3.05	0.15	.002
*s*(*υ*_0*t*_, *υ*_1*t*_)	0.06	0.81	0.07	.418
*s*(*υ*_0*t*_, *υ*_2*t*_)	-0.07	-0.95	0.08	.343
*s*(*υ*_0*t*_, *υ*_3*t*_)	-0.22	-1.96	0.11	.050
*s*(*υ*_1*t*_, *υ*_2*t*_)	0.09	1.57	0.06	.116
*s*(*υ*_1*t*_, *υ*_3*t*_)	-0.14	-1.86	0.08	.063
*s*(*υ*_2*t*_, *υ*_3*t*_)	-0.08	-0.97	0.08	.332

*I*_1*it*_ and *I*_2*it*_ are Indicator or Dummy variables indicating the stimulus category (*I*_1*it*_: 1 = African infants, 0 = Caucasian infants; *I*_2*it*_: 1 = dog puppies, 0 = Caucasian infants). *C*_*it*_ represents cuteness category (*C*_*it*_: 0 = less cute, 1 = cute). Interactions are *I*_1*it*_ *C*_*it*_ and *I*_2*it*_ *C*_*it*_. Robust estimators were used for statistical inference with respect to fixed effects and variance components to account for possible violations of model assumptions, such as normality of Level-2 residuals. Degrees of freedom were computed based on the Satterthwaite’s Approximation to account for the moderate sample size at Level 2 [46]. Therefore, the degrees of freedom were not necessarily integers and could vary across tests independent of the number of parameters.

To test the difference in mean cuteness scores between African infant and dog puppy faces, we recoded the Dummy variables and calculated a second model. In Model 2 the predictors were two Indicator variables reflecting whether the faces were African infants or not (*I*_1*it*_: African = 0, Caucasian = 1, dog puppies = 0; *I*_2*it*_: African = 0, Caucasian = 0, dog puppies = 1), cuteness category (*C*_*it*_: 0 = less cute, 1 = cute), and interactions between cuteness category and the two Indicator variables (same Equation as for Model 1). As in Model 1, we found significant differences between cute and less cute infant and puppy faces and no effect for face category (African, Caucasian, dog puppies). See Table 3 for more details. To further quantify the cuteness of each infant face we applied a method described by Glocker et al. [2]. We measured head length (hl) face width (fw), forehead length (fol), face length (fal), eye width (ew), nose length (nl), nose width (nw), and mouth width (mw) to quantify the baby schema (see Table 4). Interestingly, we found expected differences between cute and less cute faces only for Caucasian infants.

**Table 3:** Results of the pre-study (Model 2).

	Coefficient	*z*	*SE*	*p*
Fixed effects				
*Level 1*				
Intercept	2.91	17.34	0.17	<.001
*I*_1*it*_	0.01	0.07	0.11	0.941
*I*_2*it*_	0.11	0.96	0.12	0.344
*C*_*it*_	2.54	16.79	0.15	<.001
*I*_1*it*_ *C*_*it*_	0.05	0.38	1.13	.701
*I*_2*it*_ *C*_*it*_	-0.12	-0.86	1.14	.391
Random effects				
*s* ^2^(*ε*_*it*_)	1.49	37.30	0.04	<.001
*s* ^2^(*υ*_0*t*_)	0.68	3.16	0.21	.002
*s* ^2^(*υ*_1*t*_)	0.16	2.32	0.07	.020
*s* ^2^(*υ*_2*t*_)	0.17	2.36	0.07	.018
*s* ^2^(*υ*_3*t*_)	0.45	3.05	0.15	.002
*s*(*υ*_0*t*_, *υ*_1*t*_)	-0.22	-2.14	0.10	.032
*s*(*υ*_0*t*_, *υ*_2*t*_)	-0.20	-1.95	0.10	.051
*s*(*υ*_0*t*_, *υ*_3*t*_)	-0.36	-2.43	0.15	.015
*s*(*υ*_1*t*_, *υ*_2*t*_)	0.07	1.25	0.06	.210
*s*(*υ*_1*t*_, *υ*_3*t*_)	0.14	1.86	0.08	.063
*s*(*υ*_2*t*_, *υ*_3*t*_)	0.07	0.92	0.07	.357

*I*_1*it*_ and *I*_2*it*_ are Indicator or Dummy variables indicating the stimulus category (*I*_1*it*_: 1 = African infants, 0 = Caucasian infants; *I*_2*it*_: 1 = dog puppies, 0 = Caucasian infants). *C* represents cuteness category (*C*_*it*_: 0 = less cute, 1 = cute). Robust estimators were used for statistical inference with respect to fixed effects and variance components to account for possible violations of model assumptions, such as normality of Level-2 residuals. Degrees of freedom were computed based on the Satterthwaite’s Approximation to account for the moderate sample size at Level 2 [46]. Therefore, the degrees of freedom were not necessarily integers and could vary across tests independent of the number of parameters.

**Table 4:** Objective measures of baby schema (cf. Glocker et al. [2]).

	fw	fol/fal	ew/fw	nl/hl	nw/fw	mw/fw
Caucasian infants						
cute	337.42	1.49	0.19	0.16	0.24	0.28
mean	322.77	1.39	0.19	0.16	0.24	0.28
less cute	308.12	1.29	0.19	0.15	0.24	0.28
African infants						
cute	331.43	1.37	0.21	0.14	0.27	0.33
mean	334.82	1.35	0.21	0.15	0.27	0.32
less cute	338.21	1.33	0.21	0.15	0.28	0.31
dog puppies						
cute	282.39	1.06	0.15	0.36	0.24	0.24
mean	291.52	1.06	0.15	0.35	0.24	0.24
less cute	300.64	1.07	0.15	0.34	0.25	0.24

Following Glocker, Langleben, Ruparel, Loughead, Gur, et al. [2] we measured head length (hl) face width (fw), forehead length (fol), face length (fal), eye width (ew), nose length (nl), nose width (nw), and mouth width (mw) to quantify baby schema. According to Glocker, Langleben, Ruparel, Loughead, Gur, et al., the following facial parameters capture baby schema: face width as an absolute measure in pixels and 5 proportion indices representing the relative size of one facial measure to another (fol/fal, ew/fw, nl/hl, nw/fw, mw/fw). The table shows the 6 parameters for each face category and for cute and less cute faces as well as for the mean across cute and less cute faces within a face category. High baby schema faces have larger fw, fol/fal, and ew/fw than low baby schema faces. By means of simple regression analyses we tested whether cute and less cute infant faces significantly differ from each other in the 6 parameters. In our sample there were significant differences in the expected direction between cute and less cute infants for Caucasian faces in face width (*p* = .018) and the ratio of forehead length to face length (*p* = .014). No other differences between cute and less cute infant faces were significant.

## Study 1: Preference for Cute Infants

The aim of Study 1 was to establish whether cute infants are preferred in a hypothetical adoption task and in a hypothetical donation task. Using the face pairs determined in the pre-study we expected that the faces that had received higher cuteness ratings would be chosen more often in the adoption and donation task. Based on Lorenz’ [1] observations we expected that this should be true when choosing from African and Caucasian infants and dog puppies.

### Methods

#### Participants

We investigated a sample of 180 volunteers (90 female, 90 male) ranging in age between 15 and 68 (*M* = 26, *SD* = 10.2). Data was collected as part of a guided research project in a methods class. Students were asked to test colleagues, friends, and family members (e.g., younger and older siblings, parents, aunts, and uncles). All perticipants reported to be of Caucasian descent. They gave verbal informed consent and for underage participants we additionally obtained written informed consent from a parent or legal guardian. Students were instructed to explain the procedure of the experiment and to ask the subjects whether they agree to participate. Participants were told that they could abort the experiment at anytime without giving any reasons. Participants were only tested if they had agreed to take part. Because the students tested family members and close friends we did not ask for written informed consent to ensure maximal anonymity. The research and consent procedure was approved by the ethics committee of the Faculty of Human Sciences of the University of Bern and conformed with the “Ethical Principles of Psychologists and Code of Conduct” of the American Psychological Association [36].

#### Stimuli

We used 40 Caucasian and 40 African infant faces and 40 dog puppy faces taken from the pre-study. The 40 faces of each ethnicity/species were paired, each pair consisting of a cute and less cute face (see previous section and Table 1 for more details). All stimuli had a size of 400 x 400 pixels and appeared at a resolution between 113.49 and 128.65 PPI. Students used their own laptops to collect the data and thus screen size and resolution varied.

#### Procedure

Participants saw either pairs of African or Caucasian infants or pairs of dog puppies, each pair consisting of one cute and one less cute face (see pre-study). Each subject underwent two experimental blocks in which they were asked to either choose the infant to whom they would rather give a toy (Task 1) or the infant which they would rather adopt (Task 2). The order of tasks was not counterbalanced, ensuring that every participant started with a less personal decision than an imagined adoption task. Each trial started with a fixation cross. After 1000 ms the fixation cross was replaced by a face pair consisting of one cute and one less cute face. The face pair remained visible until the participants chose the left or right infant face by pressing either the “F” or “J” key on a keyboard (“F” to choose the left face and “J” to choose the right face) with their respective index fingers. Each face was presented once per task. We balanced the side on which each face was presented across participants and used different face pairings in the first and the second block to ensure that each decision was a new one. We controlled for cuteness difference between face pairs. All participants saw the same pairs within one face type (Caucasian, African, or puppies) in order to keep possible pair effects constant across participants, but face pairs were counterbalanced across tasks.

### Results

We analyzed the data using binary multilevel regression analyses for repeated measurements [37], enabling us to analyze the response of each participant to each face pair instead of having to average the decisions and calculating proportion correct across face pairs. The dependent variable was the decision for either the cute or less cute face (1 = cute face was chosen, 0 = less cute face was chosen). For each task we analyzed the data in 3 steps. Firstly, the likelihood of the cute face being chosen was tested against 0.5 (chance level) across all conditions. Secondly, the probability of the cute face being chosen was tested against 0.5 for each face type (Caucasian, African, puppy). Finally, a complex model including face type, participants’ sex and age were used to predict the probability of the cute face being chosen.

#### Toy-task

**Fig 1:**
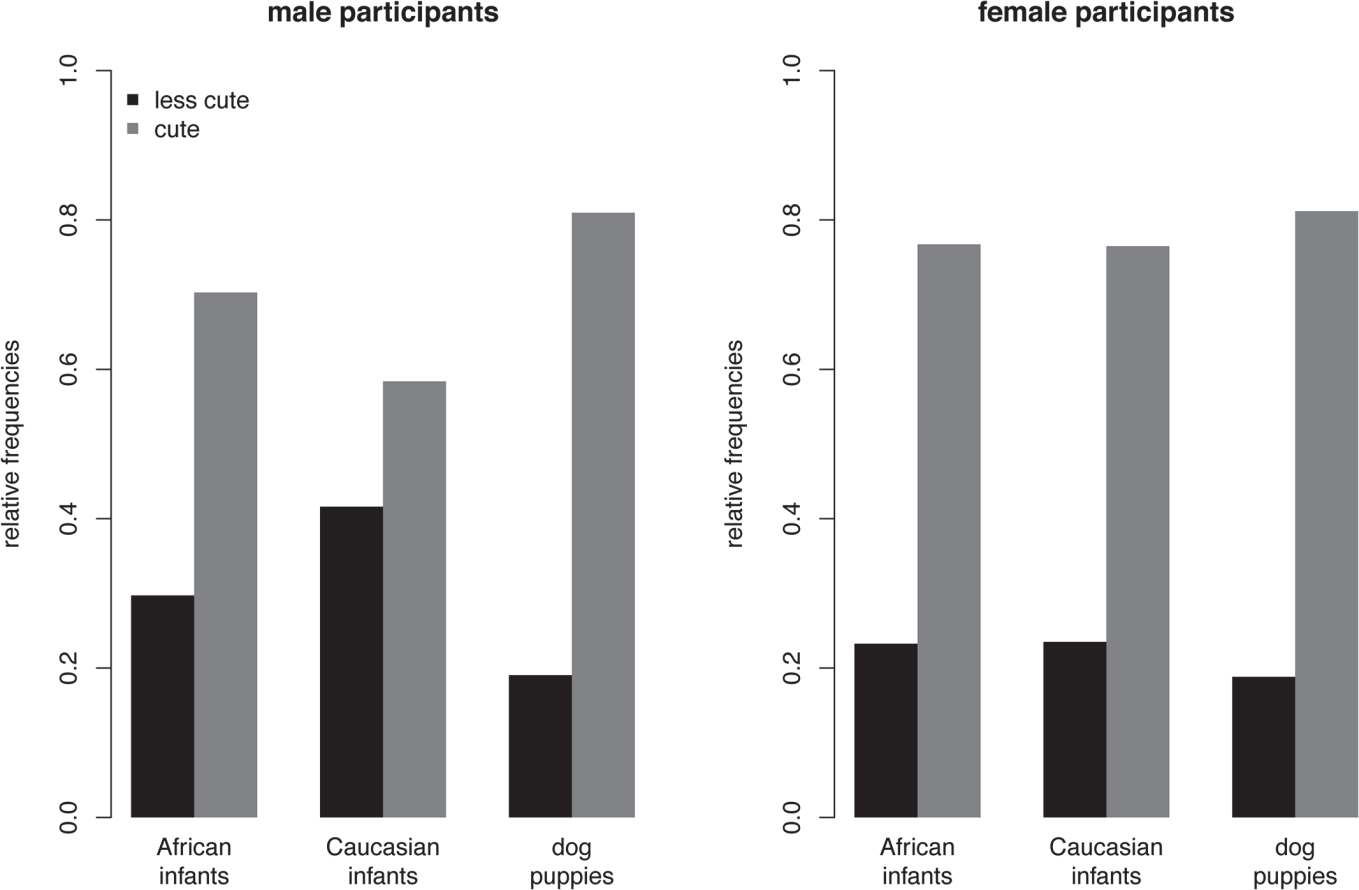
Descriptive statistics of Study 1: Toy-Task. Relative frequencies are presented for cute and less cute faces being chosen for each stimulus category and separately for male and female participants.

The descriptive statistics are shown in Fig. 1. The random intercept model without predictors (null-model) revealed a significant proportion of inter-individual variance in decisions across all participants, *s* ^2^(*β*_0*t*_) = 0.94, *z* = 6.97, *SE* = 0.13, *p* < .001. Pseudo *R* ^2^ of inter-subject variance was. 22, calculated with the following formula: *s* ^2^(*β*_0*t*_)/(*s* ^2^(*β*_0*t*_)+*π* ^2^/3) [38, 39], legitimating a multilevel regression analysis. The fixed intercept of the null-model was 1.18, *z* = 14.09, *SE* = 0.08, *p* < .001, indicating that the probability of the cute face being chosen was significantly larger compared to the probability of the less cute face being chosen across all conditions and participants (i.e., the probability of the cute face being chosen is larger than 0.5).

To test whether the probability of the cute face being chosen within each face type significantly differs from 0.5 we separately calculated a null model for Caucasian infants, African infants, and dog puppies. The fixed intercepts were 1.14 (Caucasian infants), *z* = 8.08, *SE* = 0.14, *p* < .001, 0.84 (African infants), *z* = 5.38, *SE* = 0.16, *p* < .001, and 1.53 (dog puppies), *z* = 13.20, *SE* = 0.12, *p* < .001, indicating that the probability of the cute face being chosen was significantly larger compared to the probability of the less cute face being chosen across all face types and participants.

The final model included no predictors on Level 1 (within-subject variables). The Level 2 predictors (between-subject variables) were two Indicator variables (Dummy variables) representing African infants (*I*_1*t*_) and puppies (*I*_2*t*_) whereas Caucasian infant faces were assigned to the reference category. We used Caucasian infants as reference because we were interested in the effects of the own ethnicity compared to another ethnicity as well as the comparison between the own and another species. Further predictors on Level 2 were participants’ sex (*S*_*t*_: 0 = male, 1 = female), grand-mean centered age (*A*_*t*_), and all possible interactions between Level 2 predictors (see Equation 2). Grand-mean centering of a variable is achieved by subtracting the mean of a variable from each individual value.

$$\begin{array}{c} L\text{og}it\left(Y_{it}\right)=\beta_{0t}\\ \beta_{0t}=\alpha_{00}+\alpha_{01}I_{1t}+\alpha_{02}I_{2t}+\alpha_{03}S_{t}+\alpha_{04}A_{t}+\alpha_{05}S_{t}A_{t}+\alpha_{06}I_{1t}S_{t}+\alpha_{07}I_{2t}S_{t}+\\ \alpha_{08}I_{1t}A_{t}+\alpha_{09}I_{2t}A_{t}+\alpha_{010}I_{1t}S_{t}A_{t}+\alpha_{011}I_{2t}S_{t}A_{t}+\upsilon_{0t} \end{array}$$ (2)

The results revealed that the probability of cute Caucasian faces being chosen was larger compared to the probability of less cute Caucasian faces being chosen for all participants, because the fixed intercept differed significantly from 0, *α*_00_ = 0.98, *z* = 4.53, *p* < .001. Furthermore, the probability of the cute face being chosen differed between the three face types. The probability of the cute face being chosen was larger for puppies, *α*_02_ = 0.60, *z* = 2.04, *p* = .043, and lower for African infants’ compared to Caucasian infants’ faces, *α*_01_ = -0.60, *z* = -2.06, *p* = .041. Men and women did not differ in their decisions, neither for Caucasian faces, *α*_03_ = 0.34, *z* = 1.17, *p* = .242, nor for faces of a different ethnicity, *α*_06_ = 0.83, *z* = 1.97, *p* = .051, or species, *α*_07_ = -0.32, *z* = -0.84, *p* = .400. Furthermore, participants’ age had no influence on the probability of the cute face being chosen, all *p*’s ≥. 305. The interactions between sex and age, and between sex, age, and the Dummy variables were also not significant, all *p*’s ≥. 335. All results are displayed in Table 5.

**Table 5:** Results of Study 1 (Toy-Task).

	Coefficient	*z*	*SE*	*p*
Fixed effects				
*Level 2*				
Intercept	0.98	4.53	0.22	<.001
*I*_1*t*_	-0.60	-2.06	0.29	.041
*I*_2*t*_	0.59	2.04	0.29	.043
*S*_*t*_	0.34	1.17	0.29	.242
*A*_*t*_	-0.01	-0.66	0.01	.512
*S*_*t*_ *A*_*t*_	<-.01	-0.01	0.03	.993
*I*_1*t*_ *S*_*t*_	0.83	1.97	0.42	.051
*I*_2*t*_ *S*_*t*_	-0.32	-0.84	0.38	.400
*I*_1*t*_ *A*_*t*_	0.02	1.03	0.02	.305
*I*_2*t*_ *A*_*t*_	< .01	0.14	0.03	.886
*I*_1*t*_ *S*_*t*_ *A*_*t*_	0.04	0.97	0.04	.335
*I*_2*t*_ *S*_*t*_ *A*_*t*_	0.02	0.50	0.04	.621
Random effects				
*s* ^2^(*υ*_0*t*_)	0.82	6.55	0.13	<.001

*I*_1*t*_ and *I*_2*t*_ are Indicator or Dummy variables indicating the stimulus category (*I*_1*t*_: 1 = African infants, 0 = Caucasian infants; *I*_2*t*_: 1 = dog puppies, 0 = Caucasian infants). *S*_*t*_ represents participants’ sex (0 = male, 1 = female). *A* indicates participants’ age (0 = mean age of the sample).

Robust estimators were used for statistical inference with respect to fixed effects and variance components to account for possible violations of model assumptions, such as normality of Level-2 residuals. Degrees of freedom were computed based on the Satterthwaite’s Approximation to account for the moderate sample size at Level 2 [46]. Therefore, the degrees of freedom were not necessarily integers and could vary across tests independent of the number of parameters.

#### Adoption-task

**Fig 2:**
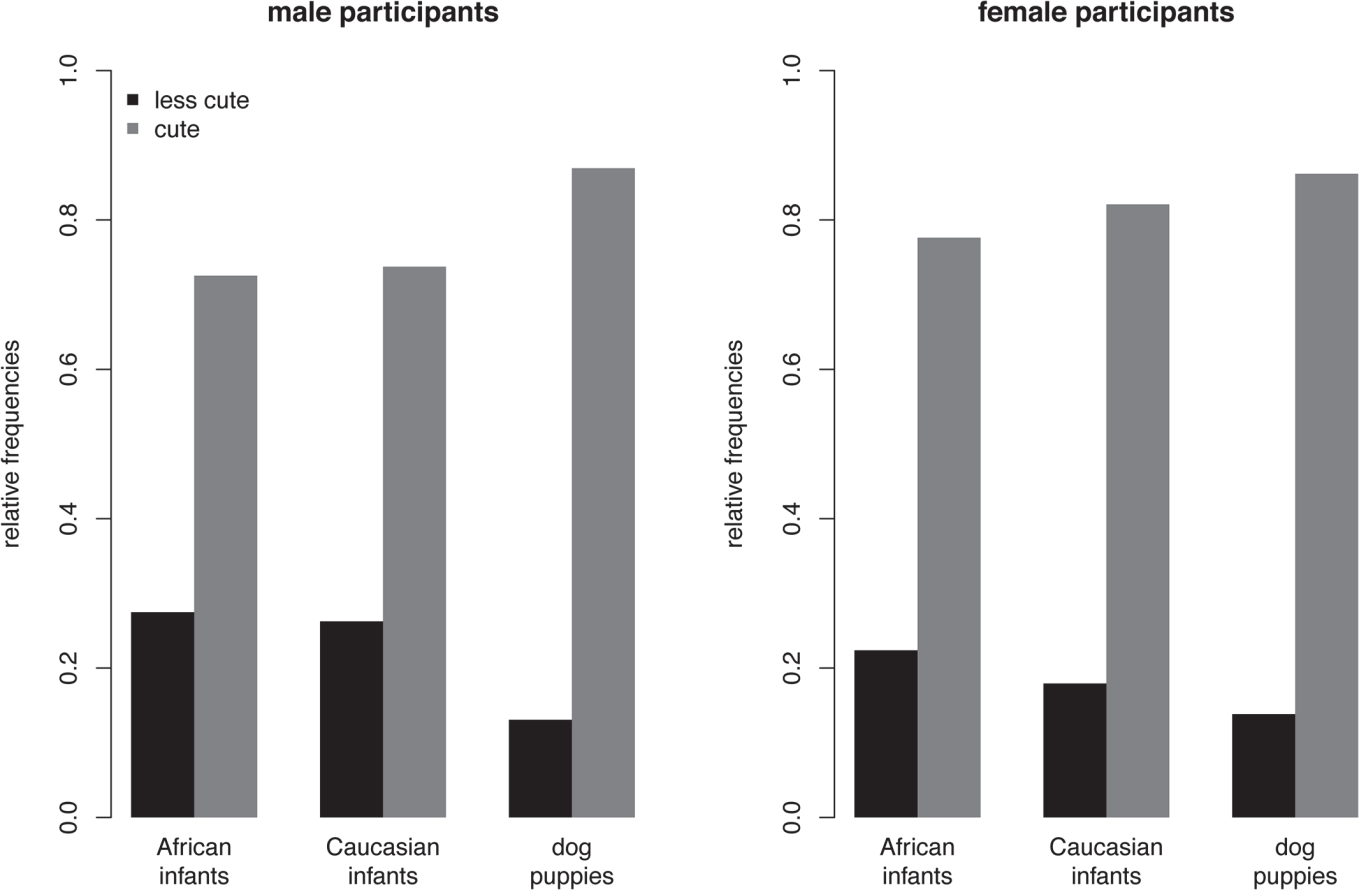
Descriptive statistics of Study 1: Adoption-Task. Relative frequencies are presented for cute and less cute faces being chosen for each stimulus category and separately for male and female participants.

The descriptive statistics are shown in Fig. 2. The null-model revealed a significant proportion of inter-individual variance in decisions across all participants, *s* ^2^(*β*_0*t*_) = 0.73, *z* = 6.39, *SE* = 0.11, *p* < .001. Pseudo *R* ^2^ of inter-subject variance was. 18. The fixed intercept of this model was 1.51, z = 19.33, *SE* = 0.08, *p* < .001, indicating that the probability of the cute face being chosen was significantly larger compared to the probability of the less cute face being chosen across all face types (Caucasian, African, puppy) and participants.

In line with the analysis of Task 1 we separately calculated a null model for Caucasian infants, African infants, and dog puppies to test whether the probability of the cute face being chosen within each face type significantly differs from 0.5. The fixed intercepts were 1.22 (Caucasian infants), *z* = 9.09, *SE* = 0.13, *p* < .001, 1.36 (African infants), *z* = 10.79, *SE* = 0.13, *p* < .001, and 1.95 (dog puppies), *z* = 15.43, *SE* = 0.13, *p* < .001, indicating that the probability of the cute face being chosen was significantly larger compared to the probability of the less cute face being chosen across all face types and participants.

The final model was the same as in the toy-task (see Equation 2) revealing similar results. Neither participants’ sex nor age had an influence on the higher probability of the cute face being chosen across all conditions, all *p*’s ≥. 098. Furthermore, the probability of the cute face being chosen was lower for Caucasian infant compared to puppy faces, *α*_02_ = 0.88, *z* = 3.03, *p* = .003. The difference between Caucasian and African infants did not reach significance, *α*_01_ = -0.01, *z* = -0.03, *p* = .976. All results are displayed in Table 6.

**Table 6:** Results of Study 1 (Adoption-Task).

	Coefficient	*z*	*SE*	*P*
Fixed effects				
*Level 2*				
Intercept	1.13	5.36	0.21	<.001
*I*_1*t*_	-0.01	-0.03	0.27	.976
*I*_2*t*_	0.88	3.04	0.29	.003
*S*_*t*_	0.21	0.78	0.28	.437
*A*_*t*_	-0.01	-1.11	0.01	.270
*S*_*t*_ *A*_*t*_	0.02	0.74	0.02	.460
*I*_1*t*_ *S*_*t*_	0.39	1.12	0.35	.266
*I*_2*t*_ *S*_*t*_	-0.29	-0.77	0.38	.443
*I*_1*t*_ *A*_*t*_	0.02	1.69	0.01	.098
*I*_2*t*_ *A*_*t*_	< .01	0.01	0.03	.992
*I*_1*t*_ *S*_*t*_ *A*_*t*_	<-.01	-0.05	0.03	.963
*I*_2*t*_ *S*_*t*_ *A*_*t*_	-0.03	-0.72	0.04	.472
Random effects				
*s* ^2^(*υ*_0*t*_)	0.65	5.95	0.11	<.001

*I*_1*t*_ and *I*_2*t*_ are Indicator or Dummy variables indicating the stimulus category (*I*_1*t*_: 1 = African infants, 0 = Caucasian infants; *I*_2*t*_: 1 = dog puppies, 0 = Caucasian infants). *S*_*t*_ represents participants’ sex (0 = male, 1 = female). *A* indicates participants’ age (0 = mean age of the sample).

Robust estimators were used for statistical inference with respect to fixed effects and variance components to account for possible violations of model assumptions, such as normality of Level-2 residuals. Degrees of freedom were computed based on the Satterthwaite’s Approximation to account for the moderate sample size at Level 2 [46]. Therefore, the degrees of freedom were not necessarily integers and could vary across tests independent of the number of parameters.

### Brief discussion

As expected, cute infants were more frequently chosen in the toy-donation task and in the hypothetical adoption task, irrespective of the ethnicity and species of the stimulus faces and independent of the sex and age of the participants. In this study we tested preferences for cute infants belonging to the same ethnicity as the observer, belonging to a different ethnicity, and to a different species. For all these faces we observed the same result: cute faces were preferred. In the toy-task the cute compared to less cute dog puppies and Caucasian infants were more strongly preferred than the African infants. In the more personally relevant decision such as in the hypothetical adoption task the infants’ ethnicity had no differential effect on the preference for cute infants. However, the influence of cuteness was more pronounced in decisions related to dog puppies compared to infants of the own species.

## Study 2: Cuteness and Health

The findings of Study 1 revealed that if cute and less cute faces of African or Caucasian infants, or dog puppies are presented, cuter faces are preferred in a hypothetical adoption and a hypothetical donation paradigm. This effect occured for faces of different ethnicities and species irrespective of the observer characteristics. A possible reason for this preference is the positive relation between cuteness and health [16, 22, 27, 29–33]. If an infant looks cute, it is assumed that it is healthy, implying that parental investment is lower. To test whether the perception of health and cuteness is related across ethnicities and species we conducted Study 2.

### Methods

#### Participants

Participants were the same as in Study 1. Study 2 was conducted immediately after Study 1. Due to storage problems the data of 45 participants could not be analyzed, resulting in 145 subjects (71 female, 74 male). Participants’ age ranged between 15 and 68 years (*M* = 26, *SD* = 10.3).

#### Stimuli

The stimuli consisted of the faces used in Study 1. All stimuli had a size of 400 x 400 pixels and appeared at a resolution between 113.49 and 128.65 PPI. Students used their own laptops to collect the data and thus screen size and resolution varied.

#### Procedure

All pictures were randomly presented in two different blocks. In the first block the task was to evaluate the health of each face on a Likert-like rating scale ranging from 1 (rarely ill) to 7 (very often ill). In the second block the task was to rate the cuteness of each infant or puppy on Likert-like rating scale ranging from 1 (not at all cute) to 7 (very cute). The presentation duration of each picture was limited by participant’s response.

### Results

As in the pre-study, we analyzed the data using multilevel regressions for repeated measurements to analyze the response of each participant to each single face instead of having to average the ratings for each infant across participants. The dependent variable was the cuteness rating score. The null-model revealed a significant proportion of inter-individual variance in cuteness evaluation across all experimental conditions, *s* ^2^(*β*_0*t*_) = 0.32, *z* = 7.85, *SE* = 0.04, *p* < .001. The intra class correlation was 0.09, *s* ^2^(*ε*_*it*_) = = 3.08, *z* = 92.88, *SE* = 0.03, *p* < .001, legitimating a multilevel regression analysis.

The predictors on Level 1 were the grand mean-centered illness rating score (*H*_*it*_), the two Indicator variables representing African infants (*I*_1*it*_) and puppies (*I*_2*it*_), and both interaction terms (*H*_*it*_ *I*_1*it*_ and *H*_*it*_ *I*_2*it*_). As in Study 1, we used Caucasian infants as reference category, enabling us to compare the effects of the own ethnicity with effects of another ethnicity as well as between the own and another species. The Level 2 predictors were participant sex and grand mean-centered age (see Equation 3).

$$\begin{array}{c} Y_{it}=\beta_{0t}+\beta_{1t}H_{it}+\beta_{2t}I_{1it}+\beta_{3t}I_{2it}+\beta_{4t}H_{it}I_{1it}+\beta_{5t}H_{it}I_{2it}+\varepsilon_{it}\\ \forall \beta_{jt}(j=0,...,5):\beta_{jt}=\alpha_{j0}+\alpha_{j1}S_{t}+\alpha_{j2}A_{t}+\alpha_{j3}S_{t}A_{t}+\upsilon_{jt} \end{array}$$ (3)

The descriptive statistics are shown in Table 7. For interpreting the following effects all other predictors have to be held constant. The results revealed that there was no significant difference in cuteness evaluation of Caucasian compared to African infants, *α*_20_ = 0.14, *z* = 1.80, *p* = .075, and no significant difference in cuteness evaluation of Caucasian infants compared to puppies, *α*_30_ = 0.04, *z* = 0.29, *p* = .773. There was also no difference in cuteness ratings given by male and female participants, *α*_01_ = 0.14, *z* = 1.11, *p* = .269. Surprisingly, participants’ age had a positive effect on cuteness evaluation. Older subjects gave higher cuteness ratings, *α*_02_ = 0.02, *z* = 2.80, *p* = .007. All results are presented in Table 8.

**Table 7:** Descriptive statistics of Study 2.

	partcipant sex	cuteness rating	health rating
Caucasian infants	male	4.01 ± 1.7	3.59 ± 1.6
	female	4.19 ± 1.9	3.53 ± 1.7
African infants	male	3.79 ± 1.7	4.24 ± 1.6
	female	3.92 ± 1.9	4.18 ± 1.6
dog puppies	male	3.86 ± 1.9	3.88 ± 1.6
	female	4.10 ± 2.0	3.89 ± 1.6

Means and standard deviations of the cuteness and health rating scores are presented. The range for each rating was 1 to 7 (not at all cute to very cute, rarely ill to very often ill).

**Table 8:** Results of Study 2.

	Coefficient	*z*	*SE*	*P*
Fixed effects				
*Level 1*				
Intercept	3.82	48.85	0.08	<.001
*H*_*it*_	-0.47	-10.95	0.04	<.001
*I*_1*it*_	0.14	1.80	0.08	.075
*I*_2*it*_	0.04	0.29	0.12	.773
*H*_*it*_ *I*_1*it*_	0.08	2.65	0.03	.009
*H*_*it*_ *I*_2*it*_	0.14	3.44	0.04	.001
*Level 2*				
*S*_*t*_	0.14	1.11	0.12	.269
*A*_*t*_	0.02	2.80	0.01	.007
*S*_*t*_ *A*_*t*_	-0.01	-0.91	0.01	.366
*Cross-level*				
*H*_*it*_ *S*_*t*_	0.01	0.21	0.01	.837
*I*_1*it*_ *S*_t_	-0.04	-0.31	0.12	.758
*I*_2*it*_ *S*_t_	0.08	0.44	0.18	.663
*H*_*it*_ *I*_1*it*_ *S*_*t*_	-0.05	-1.10	0.04	.273
*H*_*it*_ *I*_2*it*_ *S*_*t*_	-0.06	-1.26	0.05	.212
*H*_*it*_ *A*_*t*_	<-.01	-0.48	< .01	.632
*I*_1*it*_ *A*_*t*_	-0.01	-1.40	0.01	.168
*I*_2*it*_ *A*_*t*_	-0.01	-1.15	0.01	.261
*H*_*it*_ *I*_1*it*_ *A*_*t*_	< .01	0.99	< .01	.326
*H*_*it*_ *I*_2*it*_ *A*_*t*_	< .01	0.88	0.01	.379
*H*_*it*_ *S*_*t*_ *A*_*t*_	< .01	0.63	0.01	.534
*I*_1*it*_ *S*_*t*_ *A*_*t*_	-0.01	-0.75	0.01	.457
*I*_2*it*_ *S*_*t*_ *A*_*t*_	-0.02	-1.02	0.02	.312
*H*_*it*_ *I*_1*it*_ *S*_*t*_ *A*_*t*_	-0.01	-1.66	0.01	.100
*H*_*it*_ *I*_2*it*_ *S*_*t*_ *A*_*t*_	-0.01	-2.30	0.01	.023
Random effects				
*s* ^2^(*ε*_*it*_)	2.17	90.95	0.02	<.001
*s* ^2^(*υ*_0*t*_)	0.45	7.35	0.06	<.001
*s* ^2^(*υ*_1*t*_)	0.11	6.82	0.02	<.001
*s* ^2^(*υ*_2*t*_)	0.38	6.11	0.06	<.001
*s* ^2^(*υ*_3*t*_)	0.98	7.44	0.14	<.001
*s* ^2^(*υ*_4*t*_)	0.02	2.00	0.01	.006
*s* ^2^(*υ*_5*t*_)	0.04	3.37	0.01	.022
*s*(*υ*_0*t*_, *υ*_1*t*_)	0.05	2.08	0.02	.037
*s*(*υ*_0*t*_, *υ*_2*t*_)	-0.14	-2.97	0.05	.003
*s*(*υ*_0*t*_, *υ*_3*t*_)	-0.37	-5.03	0.07	<.001
*s*(*υ*_0*t*_, *υ*_4*t*_)	-0.02	-0.92	0.02	.359
*s*(*υ*_0*t*_, *υ*_5*t*_)	-0.01	-0.34	0.02	.734
*s*(*υ*_1*t*_, *υ*_2*t*_)	-0.07	-3.21	0.02	.001
*s*(*υ*_1*t*_, *υ*_3*t*_)	-0.02	-0.53	0.03	.599
*s*(*υ*_1*t*_, *υ*_4*t*_)	-0.03	-2.95	0.01	.003
*s*(*υ*_1*t*_, *υ*_5*t*_)	-0.01	-0.97	0.01	.332
*s*(*υ*_2*t*_, *υ*_3*t*_)	0.07	1.09	0.06	.278
*s*(*υ*_2*t*_, *υ*_4*t*_)	0.01	0.38	0.02	.706
*s*(*υ*_2*t*_, *υ*_5*t*_)	0.01	0.28	0.02	.783
*s*(*υ*_3*t*_, *υ*_4*t*_)	-0.02	-0.65	0.03	.515
*s*(*υ*_3*t*_, *υ*_4*t*_)	<-.01	-0.06	0.03	.955
*s*(*υ*_4*t*_, *υ*_5*t*_)	<-.01	-0.07	0.01	.948

*I*_1*it*_ and *I*_2*it*_ are Indicator or Dummy variables indicating the stimulus category (*I*_1*it*_: 1 = African infants, 0 = Caucasian infants; *I*_1*it*_: 1 = dog puppies, 0 = Caucasian infants). *H*_*it*_ reflects the health state (0 = mean assessment of perceived health across all stimuli and participants, a positive value indicates perceived above-average illness frequency). *S*_*t*_ represents participants’ sex (0 = male, 1 = female). *A*_*t*_ indicates participants’ age (0 = mean age of the sample). For interpreting the coefficients all other predictor variables have to be held constant.

An unstructured covariance structure was used for the random part at Level 2. Hence, the variances and covariances of Level 2 residuals were estimated without any constraints. Robust estimators were used for statistical inference with respect to fixed effects and variance components to account for possible violations of model assumptions, such as normality of Level-2 residuals. Degrees of freedom were computed based on the Satterthwaite’s Approximation to account for the moderate sample size at Level 2 [46]. Therefore, the degrees of freedom were not necessarily integers and could vary across tests independent of the number of parameters.

As expected, the cuteness rating score was closely associated with the mean centered illness score, *α*_10_ = -0.47, *z* = -10.95, *p* < .001, indicating that the higher the perceived illness, the lower the cuteness was rated. Furthermore, the interactions between illness and the Indicator variable 1 (African) and 2 (puppies) reached statistical significance, *α*_40_ = 0.08, *z* = 2.65, *p* = .009 and *α*_50_ = 0.14, *z* = 3.44, *p* = .001, reflecting that the negative association between perceived illness and cuteness is weaker for African infants and dog puppies compared to Caucasian infants.

Furthermore, there was a significant 4-way interaction between health, sex, age, and Dummy variable 2, *α*_53_ = -0.01, *z* = -2.30, *p* = .023 indicating that for older females the association between cuteness and health for faces of a different species compared to the own ethnicity was stronger than for male participants. For younger participants the sex effect was less pronounced. No other effects were significant.

### Brief discussion

The results revealed that perceived illness has a negative influence on perceived cuteness irrespective of ethnicity and species. Moreover, we observed a differential effect of cuteness on perceived health depending on the ethnicity and species of the faces. For human participants the strongest association between perceived health and cuteness was within the own ethnicity.

## General Discussion

In the present research we investigated preferences for cute infant faces of different ethnicities and different species in two different tasks (Study 1). Using a 2 alternative forced-choice paradigm, participants chose the infant to whom they would rather give a toy and which infant they would prefer to adopt. We found that cuter infants were generally more frequently chosen. In a subsequent study we examined the relationship between perceived cuteness and perceived health (Study 2). Here we found that perceived frequency of illness was negatively associated with cuteness. The findings of both studies were comparable for faces of African and Caucasian infants and dog puppies.

This is the first study revealing that cute infants are preferred even if these infants belong to another ethnicity or species than the observer. This preference was evident in a task where participants were asked to choose the infant to whom they would rather give a toy and in a hypothetical adoption task. We found that men and women across various ages responded in a similar way, suggesting that cues of infant cuteness are perceivable for both men and women and influence personally relevant altruistic decisions.

The findings of this study underline the universality of infant cuteness originally described by Konrad Lorenz [1], [see also 23, 24]. Previous work showed that altering paedomorphic characteristics such as enlarging the eyes or changing the height of the forehead influences preferences and decisions [e.g. 2, 3]. We extended these findings by showing that subjective cuteness evaluation of natural, unmanipulated infant faces influences choices in a hypothetical adoption and toy-donation task. At least in humans, subjective cuteness evaluations seem to trigger pro-social behavior patterns independent of the infant’s ethnicity and even independent of its species [see also the findings of 25, 26].

According to the results of Study 2, the association between perceived health and cuteness may possibly account for this finding. For adults it has often been suggested that attractiveness is a sign of good genes and health [especially in female faces, e.g. 40, 41]. Yamamoto et al. [29] found evidence of a similar relationship between infant cuteness and health: healthy infants were rated to look cuter than infants suffering from Down’s syndrome or fetal alcoholic syndrome. Furthermore, Volk and colleagues [16, 22] found a positive association between perceived cuteness and health across healthy Asian and Caucasian infants. We extended these findings by comparing cuteness and illness ratings of Caucasian and African infants and dog puppies. Specifically, we compared the magnitude of the health-cuteness association for infants of the own (Caucasian) and a different ethnicity (African) as well as for infants of a different species (dogs). For all stimulus classes we found a negative association between perceived frequency of illness and cuteness, but the association was strongest for infant faces of the own ethnicity. A potential explanation might be that although cuteness is a universal facial characteristic it is more common to invest and breed offspring of one’s own kin, that is offspring that shares genes and physical characteristics. Infants belonging to the own ethnicity naturally share more common characteristics. Importantly, different baseline levels of cuteness do not explain this finding because we found no overall difference in cuteness evaluation between Caucasian and African infants and Caucasian infants and puppies. Taking into account the results of Study 2, the preferences for cute infants found in Study 1 are potentially explained by smaller costs for raising a cute compared to a less cute infant because cute babies seem healthier.

None of our studies revealed systematic differences between male and female observers. This is in contrast to various studies reporting sex differences in the processing of cuteness and in reactions towards infants [15, 19, 29, 34, 35, 42–45]. For instance, compared to men, women smile more when seeing a cute baby [19], they extend viewing time when cute faces are presented [34, but see 43, 45], and they are more sensitive towards cuteness differences in infant faces [15]. No sex differences are reported for cuteness evaluation [19, 43], and emotional responses towards infants [19]. Furthermore, Golle et al. [23, Exp.^1^] did not find evidence for a different coding mechanism of cuteness in male and female participants. The results of the present study further support the assumption that men and women generally perceive cues of cuteness in a similar way, and that cuteness positively influences both sexes.

Surprisingly, we found a significant influence of participants’ age on cuteness evaluation and a high-order interaction between participants’ age, sex, infant face category, and perceived health. These findings might be explained by different lifetime experiences with children or age-related differences in cuteness perception and processing [cf. 35]. To the best of our knowledge there are no studies that have systematically investigated the influence of participants’ age and cuteness evaluation. Future research is necessary to shed light on these effects.

In this study we used natural portraits on human infants and dog puppies. By asking people to assess the cuteness of a series of faces, we compiled two sets of infant faces for each category: faces that were perceived as being particularly cute and faces that were perceived as being less cute. As expected, people highly agreed on which faces were cute and which ones were less cute. To further quantify the cuteness of each infant face we applied anthropometric measures to our natural stimuli [cf. 2]. Interestingly, we found expected differences in “babyfacedness” only between cute and less cute Caucasian infants. For African infants and dog puppies the anthrompometric features of faces that were rated to be very cute and faces that were rated as being less cute did not systematically differ. So, while the method used by Glocker et al. [2] seems to be useful to parametrically manipulate the cuteness of infants, it seems less advantageous to quantify cuteness in natural (unmanipulated) baby faces, especially when including faces of different ethnicities and species. As Glocker et al. [2] themselves conceded, the features of a natural face may vary in objective cuteness measurements. That is, unmanipulated infants often combine some high and some low baby schema features. We believe that using natural, unmanipulated photographs is a more ecologically valid approach than using parametrically manipulated images because, rather than being interested in specific babyfacedness measurements or in describing the features that constitute a cute face, we were interested in actual cuteness perception and in what ways cuteness perception might influence behavioural intentions (i.e., hypothetical adoption and donation of a toy).

There are some limitations of the present research. First, the stimuli we used were not controlled for sex and age. Second, we only used stimuli with a large cuteness difference in Study 1. If we had presented face pairs with smaller cuteness differences the forced choice task would have been more difficult which would have possibly lead to different results (e.g., gender differences might be observed). Third, we draw conclusions about cuteness preferences across ethnicities and species while investigating only Caucasian participants. We also note that we can not exclude the possibility that our findings are based on in- and out-group differences. Furthermore, we did not control for participants’ experiences neither with faces of African infants nor with faces of dog puppies. Future research is necessary to establish whether our findings are transferable across age and sex of infant faces, whether they are transferable to participants of other ethnicities, and to find out whether it is an ethnicity or in-group and out-group phenomenon. Despite these limitations we are able to draw conclusions about choices and ratings of naturalistic infant face stimuli varying in perceived cuteness, ethnicity, and species. Across infant faces of various natural categories and different tasks we found a preference for the cute ones and a significant association between perceived health state and cuteness evaluation. This relationship between cuteness and health may account for general cuteness preferences.

In conclusion, we found that cute infant faces belonging to the same or a different ethnicity and species than the observer were generally preferred in a hypothetical donation and adoption paradigm. We found these preferences in faces that naturally varied in perceived cuteness rather than in faces that were artificially manipulated to look more or less cute. There were no systematic differences between men and women across a wide range of ages. These findings provide further evidence for the cute-is-good stereotype and expand on previous findings about the universality of this stereotype. A strong association between perceived health and cuteness might explain the results. Irrespective of ethnicity or species, fostering cute infants might implicate that they are healthy and that therefore the costs for care-taking behavior might be lower compared to caring for less cute and potentially less healthy individuals.

## Supporting Information

S1 Dataset Dataset of the pre-study. (TXT)

S2 Dataset Dataset of Study 1: Toy-Task. (TXT)

S3 Dataset Dataset of Study 1: Adoption-Task. (TXT)

S4 Dataset Dataset of Study 2. (TXT)
